# Interdisciplinary applications of human time use with generalized lexicons

**DOI:** 10.1371/journal.pone.0270583

**Published:** 2022-07-14

**Authors:** Eric Galbraith, William Fajzel, Shirley Xu, Veronica Xia, Elena Frie, Christopher Barrington-Leigh, Victoria Reyes-García

**Affiliations:** 1 Department of Earth and Planetary Science, McGill University, Montreal, Quebec, Canada; 2 Institut de Ciència i Tecnologia Ambientals (ICTA-UAB), Universitat Autònoma de Barcelona, Cerdanyola del Vallès, Barcelona, Spain; 3 Institute for Health and Social Policy, McGill University, Montreal, Quebec, Canada; 4 Bieler School of the Environment, McGill University, Montreal, Quebec, Canada; 5 Institucio Catalana de Recerca i Estudis Avançats (ICREA), Barcelona, Spain; Menoufia University, EGYPT

## Abstract

Time use studies quantify what people do, over particular time intervals. The results of these studies have illuminated diverse and important aspects of societies and economies, from populations around the world. Yet, these efforts have advanced in a fragmented manner, using non-standardized descriptions (lexicons) of time use that often require researchers to make arbitrary designations among non-exclusive categories, and are not easily translated between disciplines. Here we propose a new approach, assembling multiple dimensions of time use to construct what we call the human chronome, as a means to provide novel interdisciplinary perspectives on fundamental aspects of human behaviour and experience. The approach is enabled by parallel lexicons, each of which aims for low ambiguity by focusing on a single coherent categorical dimension, and which can then be combined to provide a multi-dimensional characterization. Each lexicon should follow a single, consistent theoretical orientation, ensure exhaustiveness and exclusivity, and minimize ambiguity arising from temporal and social aggregation. As a pragmatic first step towards this goal, we describe the development of the Motivating- Outcome- Oriented General Activity Lexicon (MOOGAL). The MOOGAL is theoretically oriented towards the outcomes of activities, is applicable to any human from hunter-gatherers to modern urbanites, and deliberately focuses on the physical outcomes which motivate the undertaking of activities to reduce ambiguity from social aggregation. We illustrate the utility of the MOOGAL by comparing it with existing economic, sociological and anthropological lexicons, showing that it exhaustively covers the previously-defined activities with low ambiguity, and apply it to time use and economic data from two countries. Our results support the feasibility of using generalized lexicons to incorporate diverse observational constraints on time use, thereby providing a rich interdisciplinary perspective on the human system that is particularly relevant to the current period of rapid social, technological and environmental change.

## 1 Introduction

All humans, throughout history, have used exactly all of the time available to them, somehow or other. Because of this universality, quantifying how that time is used can provide a uniquely comprehensive vantage point on diverse aspects of the human system. Characterizing the distributions of states over time has been variously referred to as studying time allocation, time budgeting, or time use. The characterization generally involves associating a subject, or group of subjects, during a given time period, with a category drawn from a list (a ‘lexicon’). These categories are most often identified as ‘activities’, even if they sometimes include the absence of what is colloquially referred to as activity, such as resting or sleeping. Because of the confusion that can arise from the conflation of terms like ‘time use’ and ‘activity’ with colloquial meanings, we propose here a new term, the ‘chronome’, to refer to the quantitative description of human states of being that is explicitly referenced to defined intervals of time.

Given that quantifying what people do is useful for many reasons, study of the chronome—under its range of pseudonyms—has long been a part of many disciplines. For example, anthropologists have quantified time allocation to learn about cultures ranging from hunter-gatherers to modern urbanites [1–5]. Similarly, sociologists have applied time use data to illuminate social structures, such as gender disparities and the patterns of everyday activities [6–9], while geographers have used travel time to study commuting behaviour [10, 11]. Meanwhile, the time spent in paid work activities forms the physical basis of market economies under the name of labour [12–16], whether it be toiling in fields or factories, managing records in banks, or serving food in restaurants. The ways in which people spend their time, including the surroundings and social contexts, have been linked to their daily experiences of life, given that activities can differ greatly in their subjective outcomes [17–22] and also have consequences for health, fitness and nutrition [23–25]. Importantly, the finite availability of time—a strict 24 h per day—places an inescapable bound on the outcomes that might be achieved by a given human population, including our ongoing adaptations to technological change, how we can develop human capabilities through learning, our capacity to deal with environmental crises, and our time to enjoy life.

However, because of its multidimensional nature, describing the state of a human at a given point in time is not straightforward. Humans undertake activities in response to complex psychological motivations, and the state of any human is influenced by technological constructs, the environment, and other people, engaging the mind and body in myriad ways. A person might describe their status in a very different way than a detached observer, based on their own reasons for doing an activity, or the subjective nature of their experience. Even more importantly, a detached observer could choose to emphasize any of a multitude of aspects, so that the same status might be described in very different terms by an anthropologist, economist, sociologist, medical researcher, or philosopher, according to disciplinary norms. For example, a national Time Use Survey (TUS) will typically include rich detail regarding what an economist simply lumps together as leisure and domestic work, while relegating any activity that is done for monetary exchange to a single category of paid work. Instead, economists focus on work that is done for pay or profit, using entirely different descriptions, such as the International Standard Industrial Classification of all economic activities (ISIC) [26]. Meanwhile, anthropologists have developed their own methods, often tailored to particular cultures and frequently focused on the interests of the individual researcher [5].

Recognition of the standardization problem within the realm of national, policy-oriented household surveys prompted the definition of the International Classification of Activities for Time Use Statistics (ICATUS) in 2016. As stated in the UN Guidelines for Harmonizing Time Use Surveys [27], there had previously been ‘no single approved international standard classification of activities for time-use surveys, which limits international comparability and impacts on the ability to achieve standardization in the collection and output of activity data’. Yet, Europe continues to use its own classification system (HETUS) while each non-European country uses its own set of activity classifications. An engaged effort has been made by the Multinational Time Use Survey (MTUS) project, now housed within The Center for Time Use Research [28], to harmonize and quality-control multinational TUS data. Nonetheless, thus far these efforts have focused mostly on industrialized countries, and there have been few attempts to harmonize sociological TUS data across all available countries [29]. More to the point of this paper, we are not aware of any prior attempt to standardize lexicons more broadly, across disciplines.

The result of this disciplinary fragmentation is a diverse panoply of lexicons, each of which is suitable for addressing a particular set of research questions or national interests, but is not readily translatable to other societies or domains of research. The consequent difficulty in combining data limits the ability to construct a holistic understanding of the global human chronome and its changes through time. For example, the background context within which paid work activities occur places a powerful constraint on overall outcomes because of the 24h per day limit, but calculating this requires combining economic data with constraints on non-work activities, such as TUS data, for which the lexicons are largely incompatible. As a result it is difficult to holistically assess how activities shift between work and non-work spheres as economies change over time, or to compare wealthy countries directly with low-income countries where a large fraction of work happens outside the formal economy. What’s more, the compression of many aspects of human status into a single set of categories, in any given study, limits the range of questions to which they can possibly be applied. The lack of interdisciplinary portability of existing lexicons has prevented the study of human status over time from achieving its full potential for revealing mechanistic links between activities and outcomes, and thereby informing complex interacting aims such as those encapsulated by the Sustainable Development Goals [30].

This paper suggests that the study of time use could become a more powerful tool by moving beyond the current state of ad hoc, fragmented lexicons to deliberately build toward an open, multi-dimensional, quantitative assessment of human status over time: a global human chronome. Functionally, the approach requires defining generalized lexicons that can be applied in parallel (i.e. simultaneously), each of which aims to be broadly applicable, captures a coherent, physically-definable dimension of human status, and is both simultaneously exhaustive (can be applied at all points in time) and exclusive (without overlap between the categories within the lexicon). By separating distinct dimensions, such as by isolating the social and environmental contexts from the physical outcomes and subjective experience of activities, multiple parallel lexicons would allow subtle aspects of time use to be identified while reducing ambiguity. We are not aware of any prior attempts to design lexicons that fulfill these criteria.

Below, we expand on the challenges involved in providing generalized descriptions of human status over time, and describe one strategy for doing so that is focused on biophysical outcomes with a pragmatic alignment to existing datasets: the Motivating-Outcome-Oriented General Activity Lexicon (MOOGAL). Section 2 describes two apparent challenges that must be considered in constructing a general lexicon. Section 3 suggests a list of criteria for general lexicons, and Section 4 describes how these were applied to construct the MOOGAL, including its development and key characteristics. Section 5 briefly discusses coding procedures that mitigate ambiguity and arbitrariness. Section 6 provides examples of the MOOGAL as applied to lexicons developed for hunter gatherer societies, economic assessments, and sociological time use surveys, as well as two example countries. Section 7 concludes the paper.

## 2 The challenges

The overarching goal is to usefully categorize what humans do throughout a given day, as objectively and reproducibly as possible, for any human society. In part, this involves challenges that are common to the universal cognitive problem of categorization [31]. In addition, the dearth of prior efforts to produce generalized time use lexicons suggests the existence of specific challenges. We identify two apparent challenges that are particular to the goal of designing universally-applicable lexicons: the need to follow a consistent theoretical orientation, and the problem of multitasking and aggregation.

### Theoretical orientation

Existing lexicons have applied many different approaches to classifying the states of humans, usually without thoroughly acknowledging the implicit theoretical orientations that underlie the definitions. We here identify three orientations that are used to differentiate between categories. Existing lexicons sometimes use one orientation to define some categories, while using another orientation to define others. Although the inclusion of inconsistent orientations may be acceptable within the context for which an individual lexicon was created, it creates problems for comparing across contexts, and makes it very difficult to apply parallel lexicons in an exclusive and exhaustive manner.

1. **Social motivation.** We use this term to refer to the diverse ways by which social constructs and cultural rules motivate the undertaking of an activity. For example, ‘paid work’ generally includes activities that are undertaken for the benefit of someone else, who is usually not a friend or family member, through a monetized exchange. ‘Unpaid work’, in contrast, is generally undertaken for the benefit of oneself or one’s family. Meanwhile, the activities of ‘volunteer work’ could be identical to those in paid work but without the monetary exchange. This type of social motivation description underpins the first-order divisions often used in economics and international development, such as the System of National Accounts [32], which starts by separating market from non-market activities, regardless of what is actually being done. For example, childcare will be classified differently whether one is taking care of their child at home (unpaid work), in a daycare (paid work) or if the child is a neighbour (volunteering). As a result, it can be awkward to compare societies with different social and economic systems using the SNA [33]. Differentiating based on social roles also frequently identifies a provider and a recipient, such as a teacher and student, even if the participation of both is essential for the activity to occur. This can lead to subdividing what are essentially multi-participant activities based on whom is being paid. Social motivations are not limited to monetized relationships such as these, but also extend to religious and other cultural mechanisms.
2. **Context.** This type of description focuses on features of the environment that shape the human experience, often including who else was present, the natural surroundings, or the human-made tools or equipment used during the activity. Common examples in time use surveys include ‘spending time with family’, ‘watching tv’, ‘using a computer’ and ‘reading a book’. Technology-based descriptions can be complicated by the potential use of multiple technologies at the same time, such as listening to recorded music while traveling by train, and are often only relevant to narrow periods of history given technological changes over time.
3. **Outcome.** These descriptions focus on the outcome of an activity, rather than the contextual state, usually in terms of changes in the objective world (which can include the physical outcomes for humans). Examples found in time use surveys include ‘preparing food’, ‘cutting wood’, ‘washing dishes’, ‘providing medical care’. These descriptions can become confused given multiple simultaneous outcomes, for example riding a bicycle can change someone’s location, provide exercise, and generate an enjoyable experience at the same time.

None of these three theoretical orientations can be thought of as the ‘best’ one. Rather, each can be useful for capturing particular dimensions of a human state, and is well-suited to its own set of research and policy questions. But the use of different orientations leads to incompatibility between dataset and, further complicating the issue, many existing lexicons combine these three types of descriptions, particularly in government time use surveys (e.g. Table 1). We argue that the consistent use of a single type of theoretical orientation within a lexicon limits arbitrariness in activity definitions, decreases ambiguity in interpretation, and helps to ensure exhaustiveness and exclusivity.

**Table 1:** Inconsistent theoretical orientations of categories in an existing lexicon. Five examples are drawn from the International Classification of Activities for Time Use Statistics (ICATUS). For each, we indicate the implied theoretical orientation(s).

Category	Implied orientation
Employment in corporations, government and non-profit institutions	Social motivation
Growing of crops for the market in household enterprises	Outcome + social motivation
Indoor cleaning	Outcome
Caring for children including feeding, cleaning, physical care	Outcome
Watching/listening to television and video	Context

### Multitasking and aggregation

People frequently describe themselves as doing more than one thing at a time, such as ‘minding children while cooking’ [34]. This might suggest that people can violate the 24-hour limitation in terms of how states can be categorized.

Yet, physical outcomes that can be achieved with the body at one time are typically described in singular terms, given the limitations that a person can only be in one place at a time, and that physical tasks tend to be limited by the ability to coordinate a set of manipulations. The latter is closely related to the limitation of the conscious mind to focus on one thing at a time, due to neurological constraints on working memory allocation [35] and attention limitation [36]. In fact, most instances of ‘multitasking’ can be considered as rapid switching between micro-tasks, as part of a continuum [37]. Consequently, it could be feasible to define categories in physical terms for which multitasking could be considered negligible, at the individual level.

Despite the minor importance of multitasking for an individual on short timescales, an observer could choose to describe activities at multiple scales, which reflect different levels of aggregation over time and/or aggregation over social units (multiple people). For example, the activities of a single person could be described in terms of their physical manifestation on a second-to-second basis. This type of approach has been applied in studies of everyday life for disability and robotics research, where individual physical movements are recorded by cameras and hierarchically coded [38]. This description is relatively unambiguous, physically-oriented and highly-detailed, and would capture sequential activities that might otherwise masquerade as true multitasking. Thus, a sequence of activities might include opening the refrigerator, pulling open a drawer, extracting a bag of carrots, taking out two carrots, closing the refrigerator, carrying the carrots to the counter, opening the drawer, extracting the peeler, etc. Alternatively, the same activity could be described at a temporally aggregated level (e.g. a 15-minute interval timescale) as part of a composite activity, ‘preparing dinner’. The importance of temporal scale becomes evident when one considers that the first scheme might never explicitly capture the fact that the overall intended outcome was to make dinner, while the second would lack all detail on what kind of dinner was made and what steps were required to do so.

Social aggregation also frequently appears within activity descriptions when they are used to capture composite activities that are distributed among people. For example, a food packing plant might have employees driving forklifts, moving bags of food, operating packaging equipment, organizing sales and purchasing, and managing personnel. These activities could be described in an aggregated sense as all being part of food packing, and indeed would typically be recorded at this level of aggregation in economic data. There is no objectively-correct level of aggregation, so the level of aggregation must be strategically chosen for pragmatic and/or theoretical reasons, and must be borne in mind when interpreting results. In future, one might conceive of including additional measures to quantify the social aggregation of activities as a parallel lexicon.

## 3 Recommendations for constructing a general lexicon

We propose that the challenges listed above can be addressed by accepting the impossibility of accomplishing everything with a single universal lexicon, and instead developing multiple parallel lexicons, each of which is built on a consistent theoretical foundation. As such, new lexicons can always be added, to capture additional dimensions of human existence and behaviour. Some time use surveys have already pursued the parallel lexicon approach, in a sense, by including well-being or technology-use questions alongside the basic activities (e.g. ATUS [39]). We here modify and supplement the general guidelines recommended for international time use surveys [27] to suggest the following list of features that any general lexicon should include:

1. a single, well-defined theoretical orientation to guide the definition of categories
2. clear and unambiguous definitions, preferably with explicit physical underpinnings
3. a hierarchical structure
4. categories that are mutually exclusive and exhaustive
5. applicability to any human throughout time
6. a strategy for addressing social and temporal aggregation
7. the provision of guidelines for coding

## 4 A general lexicon: The MOOGAL

This section describes how we developed a general lexicon, the MOOGAL, designed to capture a dominant axis of variation in human activity as reflected in anthropological, economic and sociological data. The MOOGAL development was guided by the recommendations laid out above. At the same time, we aim for sufficient consistency with the categories already described in existing global datasets to easily relate to the wealth of existing data on time use found in national and international statistics, as well as the academic literature. Ensuring consistency demands a certain amount of pragmatic flexibility with respect to ideal theoretical aims.

To develop a set of categories that is consistent with this strategy, we first performed a sorting of all the activities that were included in a selection of four existing lexicons. With the aim of capturing a diverse range of activities, we chose ICATUS (number of activities *n* = 166) as the international standard for national time use statistics, ISIC (*n* = 90) as the international standard of economic activities, the time use survey of India (*n* = 155) given its thorough treatment of subsistence and informal economic activities, and the Cross-Cultural Studies in Time Allocation lexicon (CCSTA, *n* = 30) as a standardized list of activities for monitoring hunter-gatherer communities. Together this provides a total of *n* = 441 activities. Review of these activities led to the adoption of three general features.

1. *Outcome orientation.* Review of the theoretical orientations underlying the 441 categories revealed that the majority identified activities in terms of their outcomes, rather than the social motivations or contextual state. We therefore made the deliberate choice to categorize according to outcome-oriented activities. In order to be more rigorous, we limited these to physical outcomes, i.e. changes that could potentially be measured in the physical state of humans and/or the world around them (whether or not these measurements are feasible at present). Physical outcomes, as intended here, include changes in neural activity and time allocation as discussed in ref. [40]. To express this concept as an equation, the fraction of time devoted to an activity *A*_*x*_ should be associable with the rate of change of one or more physical state variables *S*_*x*_, whenever possible, for example as
  $$\begin{array}{c} \frac{dS_{x}}{dt}={\left(A_{x}N\right)}^{a}C+\Gamma_{x} \end{array}$$ (1)
  where *N* is the number of humans in the population, the exponent *a* captures nonlinearity such as diminishing returns [41], *C* is a multiplier defined by the context (and which may include the influence of other state variables), and Γ_*x*_ represents all other processes that act on state variable *S*_*x*_. State variables *S*_*x*_ might include a mass of lumber, a floor area of residential buildings, an arrangement of objects in a home, or a weighted distribution of synapses. The relevant factors in the contextual multiplier *C* would vary with the activity and state variables, but could include factors such as the availability of machinery, the neural structure of the humans carrying out the activity, and features of the environment. The orientation towards physical outcomes is intended to reduce ambiguity, limit arbitrariness, and hone precision, while the deliberate link to rates of change in physical variables is intended to help conceptualize and elucidate system dynamics. The overarching structure of human-Earth state variables outlined in [40] is used as a guiding framework.
2. *Motivation focus.* Given that a single activity can have simultaneous physical outcomes on numerous state variables, we avoid ambiguity by labeling the activity according to the set of outcomes that most strongly motivates the undertaking of the activity. In other words, an activity is categorized according to the set of physical outcomes *without which the activity would not be undertaken*. In the case of multiple physical outcomes, a three tier prioritization scheme is applied (further detailed below).
3. *Aggregation inclusion.* The level of aggregation is chosen for operability with the activities recorded in available datasets. Time use survey data tend to be recorded at the individual level in 15 minute intervals using time use diaries [20]. Anthropological data are similar. Economic activity, in contrast, is recorded at the level of firms, or broad industry-level categorizations, and therefore tends to include composite activities among many people, recorded as weekly or quarterly activities. Thus, in order to be broadly consistent, the MOOGAL categories should be equally applicable to individual or collective actions, at aggregation scales up to and including firms or industry-level categorizations, and at time intervals of 15 minutes or longer. The relevant physical outcome for socially-aggregated activities is therefore that which compels the social aggregation to occur, rather than the individual motivations of the participants. Importantly, this eliminates the motivation of monetary gain, even though it is the most common motivating outcome of economic activity at the individual level—the monetary exchange can be thought of as a communication mechanism that links the individual motivations to the overall motivating outcome.

Each of the 441 activities was then printed on a small piece of paper, all papers were randomized, and then each paper was placed on a 2-dimensional plane in a distribution that clustered the motivating physical outcomes of the activities. Activities that did not have identifiable physical outcomes (e.g. ‘volunteering’, ‘paid work’) were removed. The placements were adjusted iteratively as new papers were added, until all papers were placed within emergent clusters that could be mechanistically linked with changes in physical state variable classes. The resulting clusters were named to provide an initial list of activity categories.

Subsequently, four coders were provided with these activity categories and asked to independently associate each activity of the initial 441 with one of the new categories. Disagreements between the coders were reviewed and discussed, and the descriptions of the activities were modified accordingly in order to remove ambiguity and maximize consistency with the theoretical basis. This process revealed the necessity for a prioritization scheme to prevent arbitrariness, as detailed below. The final list of 24 subcategories, with brief descriptions are given in Table 2. These subcategories can themselves be naturally aggregated to form what we refer to as eight high-level categories.

**Table 2:** MOOGAL categories.

Category	Subcategory	Description
Food provision	Food growth and collection	All activities related to the growth of edible organic matter, and/or its collection and initial storage. Includes farming, fishing, aquaculture, gathering and hunting.
	Food processing	Processing of food after collection and initial storage, by physical and chemical transformation of edible components, to prevent spoilage, detoxify, and/or facilitate transport and later use.
	Final preparation of food	Final preparation of food within days or hours of eating, including at home, restaurant, street food, catering etc. Includes cleanup of preparation surfaces, serving, and washing of dishes.
Non-food provision	Extraction of materials	The extraction of substances to be used for the creation of artifacts, buildings and infrastructure. Includes short-range transportation and stockpiling, and initial, essential processing of raw materials (required to bring the material to the most basic state that could then be used for any purpose). Also includes managing the growth of plants and animals for materials.
	Provision of energy from non-living sources	Extraction and transport of energy carriers, including construction and operation of energy transformation facilities and long-distance transportation infrastructure.
Transformation	Artifact creation and maintenance	All activities involved in creating and maintaining movable objects from raw materials (not buildings and infrastructure). Does not include minor transformation of objects during their use (e.g. writing on paper).
	Building creation and maintenance	The making and integral maintenance of any kind of building or monument, including the initial design, construction and renovation.
	Infrastructure creation and maintenance	The engineering, construction and maintenance of persistent infrastructure to transport people, materials, and information, but not energy. Includes communications infrastructure.
Maintenance of surroundings	Cleaning surfaces/textiles and arranging inhabited environment	Maintenance of living and nonliving features of inhabited space, including home and workspace interiors, grounds, decorative gardening and domestic animal care (not for eating), as well as laundry / clothes / textile washing and care.
	External waste management	Waste management that occurs outside of inhabited buildings and their immediate environment, including sewage systems and solid waste disposal / recycling.
Neural restructure	Teaching and learning	All deliberate education and research activities not incorporated as part of another activity, including going to classes, homework, teaching classes, tutoring, as well as informally educating children, purposeful story telling, and research in the academic or private sector. Does not include apprenticeships or on-the-job training.
	Religious	Religious practice and religious social/cultural events.
Somatic maintenance	Sleep	Sleeps, naps, sleeplessness.
	Hygiene and grooming	Maintaining the cleanliness and appearance of the soma through activities such as washing, dressing, cutting hair/nails, and voiding wastes. Includes personal hygiene and grooming of oneself, grooming others, and being groomed.
	Physical Childcare	Physical and practical care of young people, including cleaning, feeding and minding young children to ensure safety. Not deliberate education/teaching or interactive play.
	Healthcare	All deliberate health care, including physical and medical support (e.g. nursing, medicalized mental health care, senior care, residential care and health/medical care of self).
Organization	Moving people	Travel that is undertaken for the purpose of changing the location of a person. Includes the time of the traveler, as well as any vehicle operator.
	Moving non-people	Moving artifacts, materials and food over distances of more than a few tens of metres. Includes stocking warehouses.
	Allocation	Altering the time allocation, and control of access to objects and spatial domains, for other humans. Includes diverse decision-making, task allocation, negotiation, discussion, and record-keeping activities.
Experience-oriented	Meals	Activities centered on eating and drinking, including associated socializing.
	Active recreation	Recreation that involves an elevated metabolic activity (including light physical activity), whether undertaken purely for neural activation or including a fitness motivation.
	Social interaction	Socializing that is not part of another activity.
	Interactive stimulation	Any other activities undertaken for the sake of experience that engage motor or linguistic output. Includes play with children.
	Passive observation	Looking/listening without engaging, i.e. neither involving interactive movement or generating written or spoken language. Can have a broad range of arousal levels, from quiet rest to watching an action movie.

### High-level MOOGAL categories

**Fig 1:**
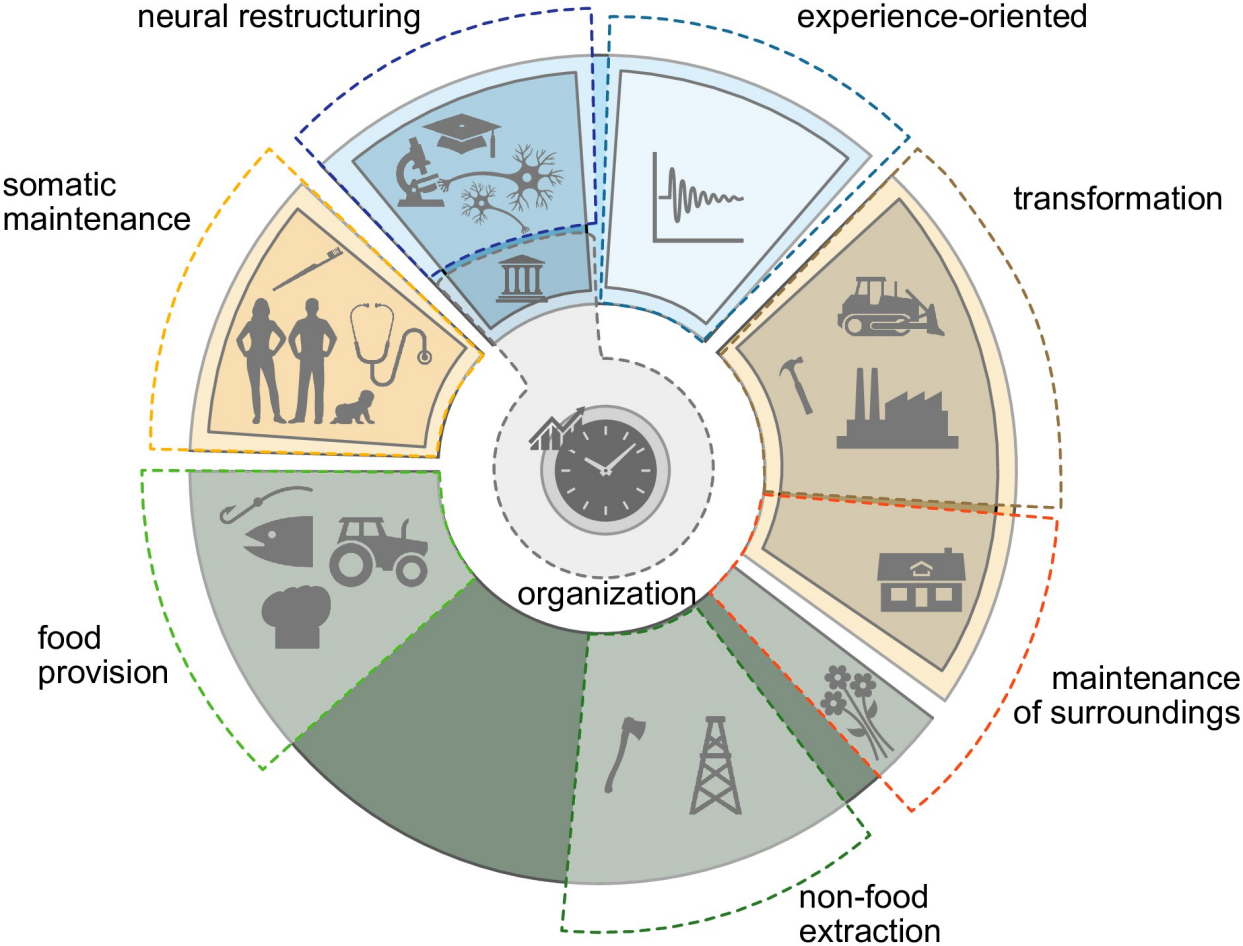
High-level MOOGAL categories. Eight categories of activities are shown schematically in relation to the human-Earth system state variables suggested by [40]. Regions with solid outlines indicate the six state variable classes of the soma (orange), neural structure (dark blue), neural activation (light blue), things (brown), time allocation (grey circle) and remainder of Earth System (green). Dashed outlines indicate relationships of MOOGAL activity categories to state variables. MOOGAL categories can each be associated with one state variable class except *maintenance of the inhabited environment*, which spans both human-created and natural spaces, and *organization*, which spans both time allocation and neural structures (via laws and other cultural norms). Note that *organization* also includes changing the locations of humans, food, materials and artifacts.

To provide an overview of the MOOGAL (version 1), the eight high-level categories are described according to their outcomes on the six state variable classes illustrated in Fig 1. The six state variable classes are designed to provide a simple conceptual superstructure for the state of the entire human-Earth system, and within which the time allocation to activities plays a central role as a determinant of rates of change, the physical outputs of activities (e.g. Eq 1). The framework explicitly considers neural structures as the physical basis of mental attributes—such as knowledge, beliefs and values—an uncommon perspective in social sciences, but one which has growing empirical support [42–44]. Similarly, experience is considered, in physical terms, as the stimulation of existing neural structures (including associated emotions and feelings) [45, 46] which occurs during all waking hours, but can also be deliberately modified by experience-oriented activities. The close alignment between physical state variables and activities is intended to facilitate the identification of mechanistic linkages within the human-Earth system.

In brief, the high-level categories are defined as follows:

1. Food provision. All activities that contribute towards the provision of food to humans. This includes anything to modify the growth of edible organisms, the collection, storage and processing of edible organisms, and the final preparation of food prior to eating (usually at home or in a restaurant) including cleanup.
2. Non-food provision. The collection of non-edible materials from the natural environment, including essential initial processing to non-specialized forms.
3. Inhabited environment maintenance. Cleaning and maintenance of surfaces and textiles, and rearrangements of objects and plants within the spaces inhabited by humans. These can include both the interiors of buildings (homes, offices, public buildings) and exterior spaces (lawns, gardens, urban parks).
4. Transformation of materials. The activities that physically transform extracted materials into the entire range of human creations (other than food), including movable objects and machines (referred to as artifacts) as well as immovable buildings and infrastructure.
5. Somatic maintenance. Care of the surface and interior of human bodies, including decorative aspects. This encompasses physical care and health care as well as personal hygiene, grooming and sleep.
6. Deliberate neural restructuring. Activities that deliberately aim to alter the neural structure of humans, including formal education, teaching through oral tradition, research and religious practices.
7. Organization. Activities that do not directly aim to achieve any of the prior categories, but which have the outcomes of a) altering time allocation through communication with others, and/or b) mediating the spatial relationships between other humans and the physical world. The latter includes the locations of people, artifacts and materials, as well as the access rights of people to locations and artifacts via their codification in neural structures and documents.
8. Experience-oriented. Activities that are motivated primarily by the subjective experience, such as meals, recreation, games, hobbies, passive observation and idle relaxation. Although oriented towards experience, activities included here may be socially-aggregated, such as socializing and participation in cultural events.

### Choices in assigning hierarchical associations

Although the definition of subcategories and their associations with high-level categories was straightforward in most cases, we were still left with the need to make a few choices in order to be able to make effective use of existing time use data. We briefly discuss the most important of these.

First, meals could be considered as somatic maintenance, given that eating is an essential part of maintaining a living body. However, breathing is also essential, and is never recorded as an activity, given its ubiquity and therefore lack of usefulness as an observation. Similarly, all living humans must eat regularly in order to stay alive, and for most humans the time spent physically ingesting food is considerably less than what is expended at meals. Meals, instead, hold a large cultural value in many societies, and are often an enjoyable activity given the sensory experience and social context. Because the meal experience typically goes far beyond the simple act of ingestion, and it is this extended experience that is likely to account for most variation in the meal time expenditure as recorded in time use surveys, the decision was made to include meals within the experience-oriented category.

Similarly, active recreation, including exercise, could be considered a somatic maintenance activity when undertaken with the deliberate aim of improving physical fitness. However, exercise is often recorded in combination with other activities that are undertaken purely for enjoyment. It is therefore included within the experience-oriented high level category.

Religious activities are diverse, ranging from individual meditation and prayer to large gatherings, rituals and social events. Because they are largely experiential, they could be categorized as experience-oriented. However, they are generally differentiated from non-religious cultural and social activities by their deliberate influences on beliefs, norms and ways of thinking, all of which involve persistent modifications of neural structure. Religious activities are therefore included within deliberate neural modification.

Maintaining built environments and their surrounding plant life could be theoretically separated, which would make it more in line with the state variables (cleanly separating buildings vs. rest of Earth system, Fig 1). However the distinction between the two domains is problematic, given the gradation from yards and gardens to public parks, and the fact that the activities are often aggregated together in existing datasets. The decision was therefore made to combine all maintenance of the inhabited environment together, including modifications of the immediate vegetation (not intended to produce food).

Finally, the organization category also produces outcomes that span two state variable classes—time use and neural structures. This was unavoidable given the fact that norms and relationships, which are encoded in neural structures, are often modified through the same activities that allocate time use in existing datasets. We also note that the transportation aspects of organization do not correspond in an obvious way with any of the state variables in ref. [40], but can be considered as modifying the location properties of soma, things and Earth system materials.

## 5 Coding procedures

Our initial trials of applying the MOOGAL to real-world lexicons identified potential ambiguities, arising from the ways in which activities are aggregated. We developed additional coding protocols to address these ambiguities, described here.

### Priority scheme

Although true multi-tasking is a rare occurrence for individuals on short timespans, as discussed above, the aggregation of activities over time and between individuals unavoidably results in the co-occurrence of multiple outcomes within the same time interval. To return to the food processing factory example above, the people involved are undertaking many organizational activities, as well as food processing, while the participants are themselves physically modified by their activities, through the neural activation stimulated by the experience as well as the outcomes on the growth of their bodies through the types of physical activity in which they are engaged. The co-occurence of simultaneous activities within a social aggregate can introduce significant ambiguity in how it should be classified.

**Fig 2:**
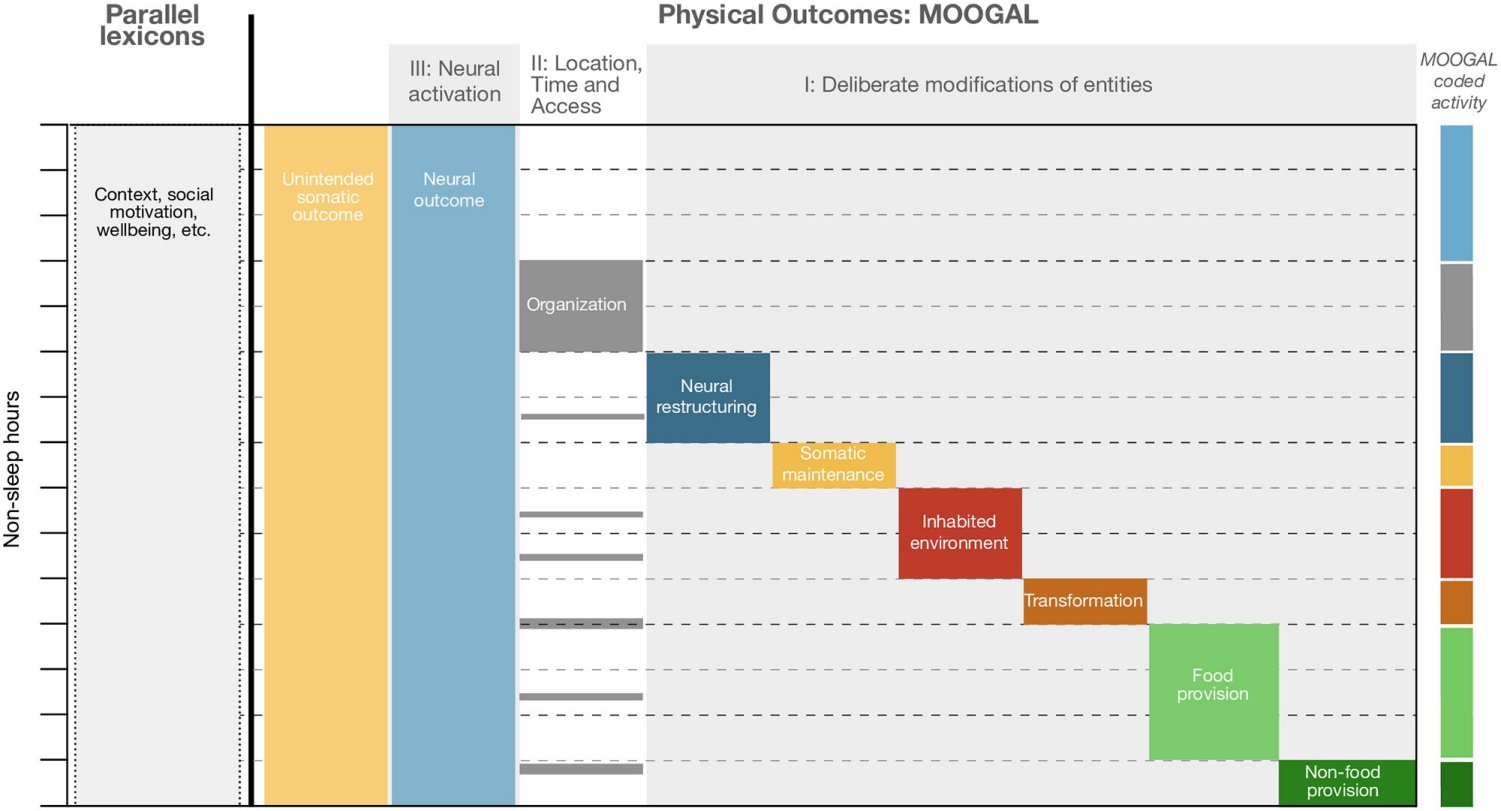
Prioritization of simultaneous outcomes. Schematic illustration of how simultaneous outcomes can occur, in relation to the prioritization scheme. Simultaneous outcomes are those which co-occur horizontally. For example, many organization outcomes (thin grey bars) could co-occur with deliberate modification activities throughout the day, but are not identified as the motivating outcomes because they are lower priority. Similarly, somatic and neural outcomes are always occurring, but somatic outcomes are only identified when they are the primary deliberate outcome, and neural outcomes are only identified when not co-occurring with one of the tier I or II outcomes. Context, social motivation and wellbeing (left-hand side) are not captured by the MOOGAL, but are examples of dimensions of time use that could be addressed with additional parallel lexicons.

To address this challenge, we introduce a priority scheme to the MOOGAL activities (Fig 2), similar to the coding priority used in the Cross-cultural Studies in Time Allocation monographs [2]. The priority scheme determines which physical outcomes of an aggregate activity are used to identify it, and gives higher priority to more easily identified outcomes. Deliberate modifications of the physical world are given the highest priority: if a compound activity has for its motivating outcome a modification of the non-human Earth system, human-made creations and environments, or humans themselves, this takes precedence over any organizational or unintended human outcomes. In the absence of a deliberate modification, organizational outcomes take precedence over neural activation (which is omnipresent) or unintended consequences for human bodies. Finally, if the activity is not undertaken to deliberately modify the world nor to produce organizational outcomes, it is identified as being undertaken for neural activation. Unintended consequences on human bodies, although always occurring, are (by definition) not motivating outcomes and are therefore never identified.

Priority is not intended to reflect a judgement regarding the normative value of activities, but simply to avoid ambiguity in the results by codifying an explicit procedure. This strategy introduces a systematic error, in that lower-priority categories will tend to be underestimated, so that the time recorded in lower priority activities should be interpreted as a lower bound. The intent of the priority scheme is to reduce arbitrariness and make results more reproducible.

### Fractional time allocation

The primary intended use of the MOOGAL is to provide a cross-mapping between the lexicons of existing datasets. These other lexicons frequently include aggregated activities that clearly span multiple motivating physical outcomes. For example, the aggregated activity ‘Housework’ (defined by social motivation and context) will typically include, among its motivating physical outcomes, some maintenance of surroundings, some food preparation, and may also include a fraction of organization in some lexicons. In order to disambiguate such aggregations, the coder uses whatever information is at their disposal (additional data, detailed descriptions, etc.) to estimate the fractional distribution of the activity between MOOGAL subcategories. The total allocation of time across the activity must sum to one, ensuring exclusivity.

The recategorization of any activity between two or more MOOGAL categories unavoidably adds a layer of uncertainty to the final results. To provide a quantitative estimate of this categorization-related uncertainty, we use a structured subjective assessment of the confidence range during the association of MOOGAL categories with the original activities, and propagate this through to the final result by weighting each category by the time devoted to each activity. The subjective confidence ranges were calculated by following three steps: 1) considering all MOOGAL categories that could conceivably be associated with an activity, according to the prioritization scheme, 2) for the dominant category, assessing the minimum and maximum feasible time fraction that it could represent (aiming for 95% confidence interval), 3) similarly assessing the feasible minimum and maximum time fractions for the remaining categories, in diminishing importance. The ranges were recorded as 1 standard error, symmetrically distributed about the mean. For example, an activity that is estimated as 60% interactive stimulation (best guess), with feasible minimum and maximum of 30% and 90%, respectively (95% confidence range) would be recorded as mean time fraction 0.6 with standard error 0.15.

## 6 Example applications

### Comparison with existing lexicons

We next show how the MOOGAL compares to three of the four lexicons introduced above: one lexicon designed for hunter-gatherer study (CCSTA), one lexicon designed for time use surveys (ICATUS), and one economic lexicon (ISIC). Four coders independently allocated each activity in these three lexicons with one or more of the 24 MOOGAL subcategories listed and briefly described in Table 2. Where an activity was identified as including multiple MOOGAL categories, this was expressed by coding time fractions between 0 and 1 (and summing to 1) using the provided descriptions of the activity components and/or supplemental information on the detailed breakdown (e.g. ISIC tier 3 subdivision). Initial disagreements between coders were discussed and resolved by consensus. The final results are shown for all activities in the Supplementary Figures and summarized here.

**Fig 3:**
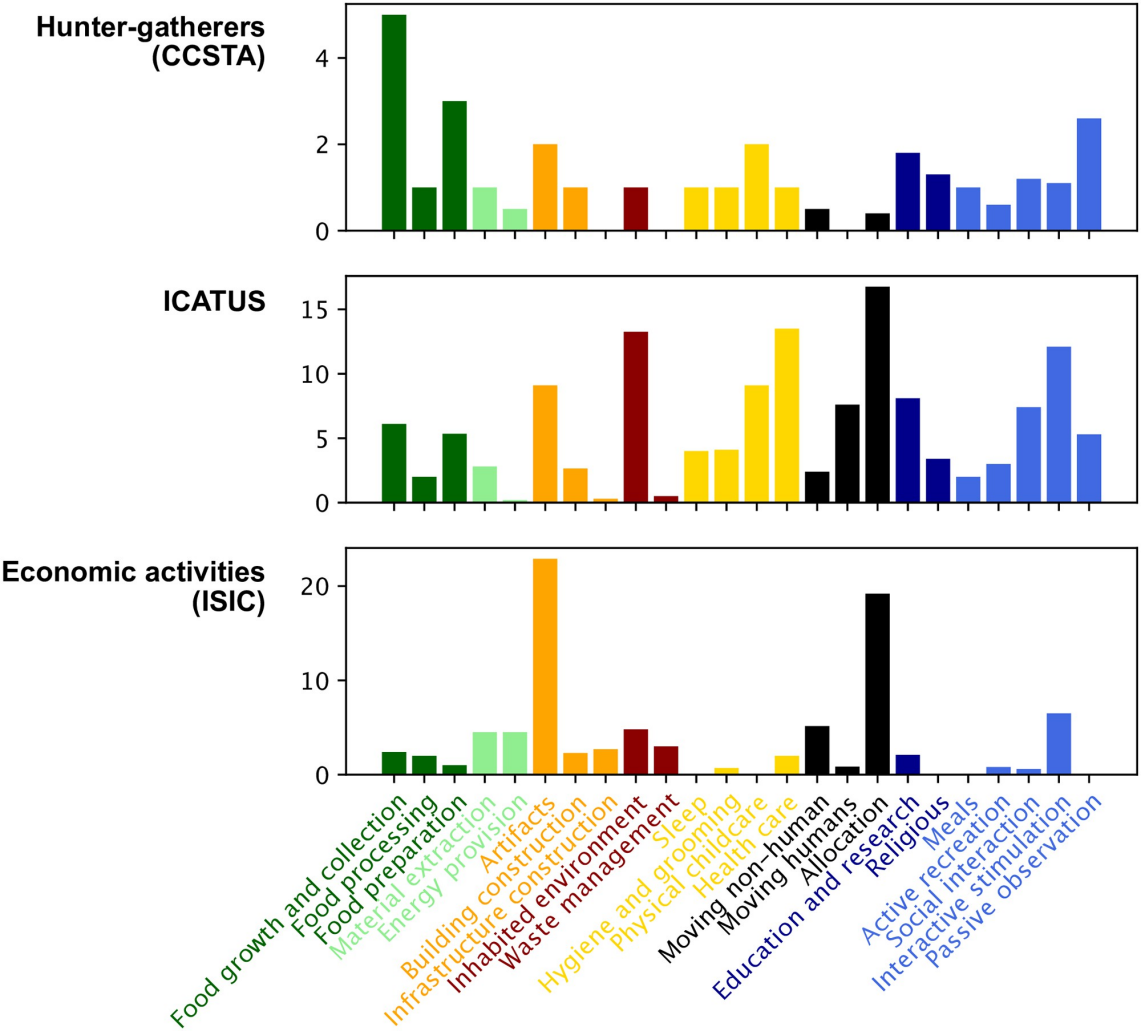
Sum of time fractions by category. Each bar shows the sum of all time fractions associated with each category, for each of the three illustrative lexicons. The sum reflects how frequently activities were associated with each MOOGAL category.

Fig 3 shows the distribution of activities across the MOOGAL categories. The distribution is more evenly-spread for the time use lexicons (both ICATUS and CCSTA), which can be explained by the fact that—like the MOOGAL—these surveys aimed at capturing largely holistic views of life. The economic (ISIC) categories are much less widely distributed across MOOGAL categories, with foci in the Artifacts and Allocation sub-categories, but still have some degree of representation across most other categories, consistent with the diverse character of economic activities. Importantly, there were no activities that could not be associated with one of the MOOGAL categories in any of the three lexicons, indicating that the MOOGAL lexicon is exhaustive. Human travel is absent from the hunter-gatherer lexicon because, by design, the lexicon is only for recording activities observed within the hunter-gatherer camp.

**Fig 4:**
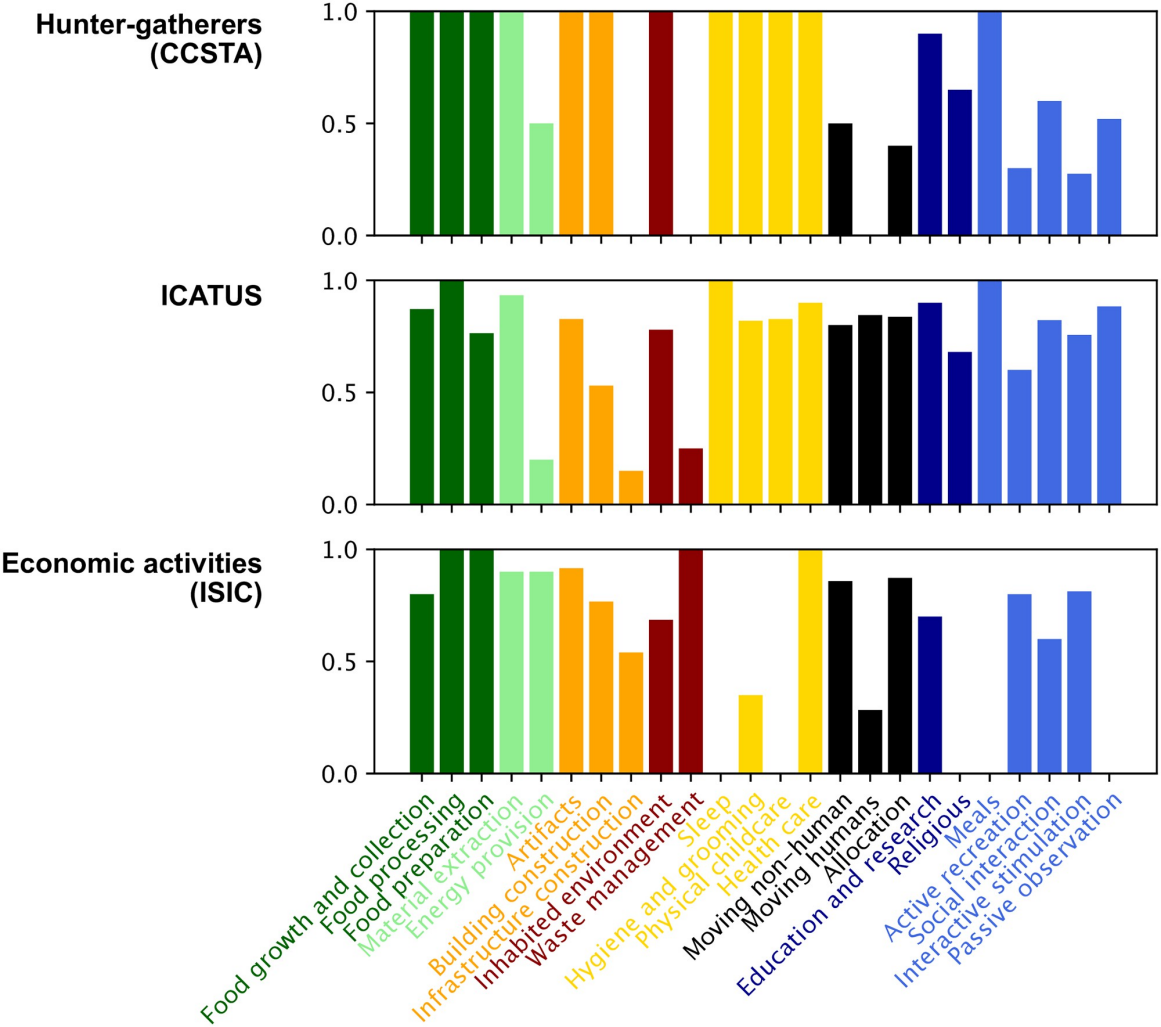
Average time fraction by MOOGAL category. Each bar shows the average of all time fractions associated with each category, for each of the three illustrative lexicons. Where the average time fraction is 1, only unambiguous, direct associations occurred within the category. Where the value is <1, the association required some subdivision of activities amongst multiple categories. For example, food processing was only unambiguously associated with activities in all three lexicons, whereas infrastructure creation and maintenance was estimated as a subcomponent of at least one activity in both ICATUS and ISIC (and is entirely absent from hunter gatherers, who did not create infrastructure).

The comparison shown in Fig 4 reveals that, although there are only 24 MOOGAL subcategories, most activities in the three larger lexicons can be directly associated entirely with one MOOGAL subcategory, indicating relatively good alignment. Each bar shows the average time fraction (ranging from 0 to 1) of all activities associated with each MOOGAL category. Where the average time fraction is 1, the corresponding MOOGAL category was unambiguously associated with one or more activities in their entirety. This direct equivalence reduces the potential for coder error and the need for subjective judgments. Where the value is less than 1, one or more activities were only partially associated with the MOOGAL category. Note that the values shown in Fig 4 cannot be directly related to uncertainty in the resulting time use calculation, since this also depends on the relative distribution of time between different activities (i.e. rare activities contribute little to the overall uncertainty).

A close examination of the supplementary figures shows that almost all of the partial time fraction designations split activities between subcategories of a high-level MOOGAL category, rather than spanning multiple high-level categories. For example, activities are frequently split between two or more experience-oriented subcategories. The only activities that are fractionally allocated between multiple high-level MOOGAL categories are some volunteer activities (ICATUS), veterinary activities (ISIC), undifferentiated goods and services production of private households for own use (ISIC), and fetching and managing household water and fuel (CCSTA). Thus, the MOOGAL associations with these three lexicons have very little uncertainty for the eight high-level categories.

### Application to two countries

**Fig 5:**
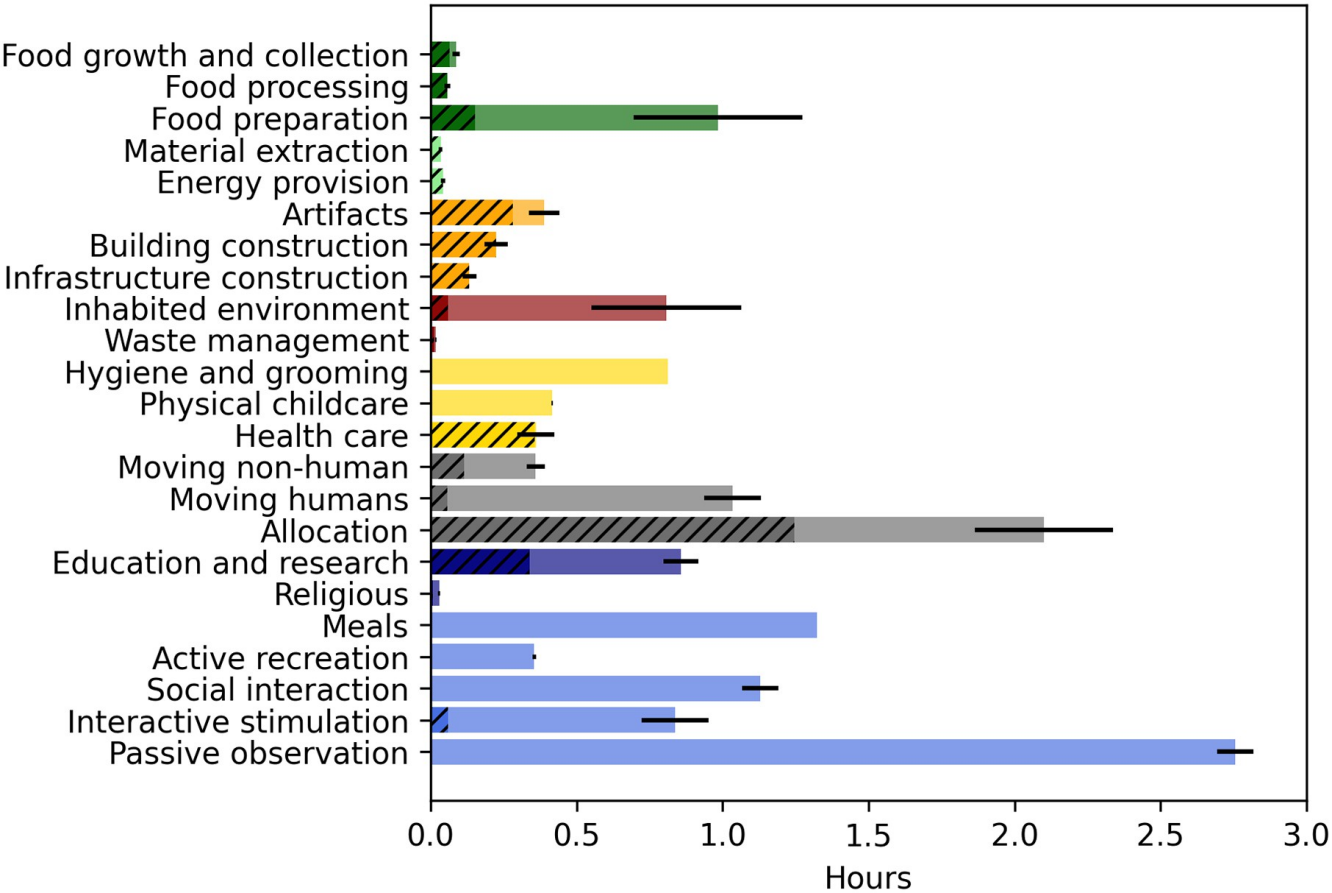
Time use in Canada using the MOOGAL. Combination of time use and economic activity data. Shaded bars show the total time allocated to each MOOGAL category, while hatched portions indicate the time allocated through the formal economy (work for pay or profit) Thin black lines indicate the 95% confidence range for the total time use in each MOOGAL category.

**Fig 6:**
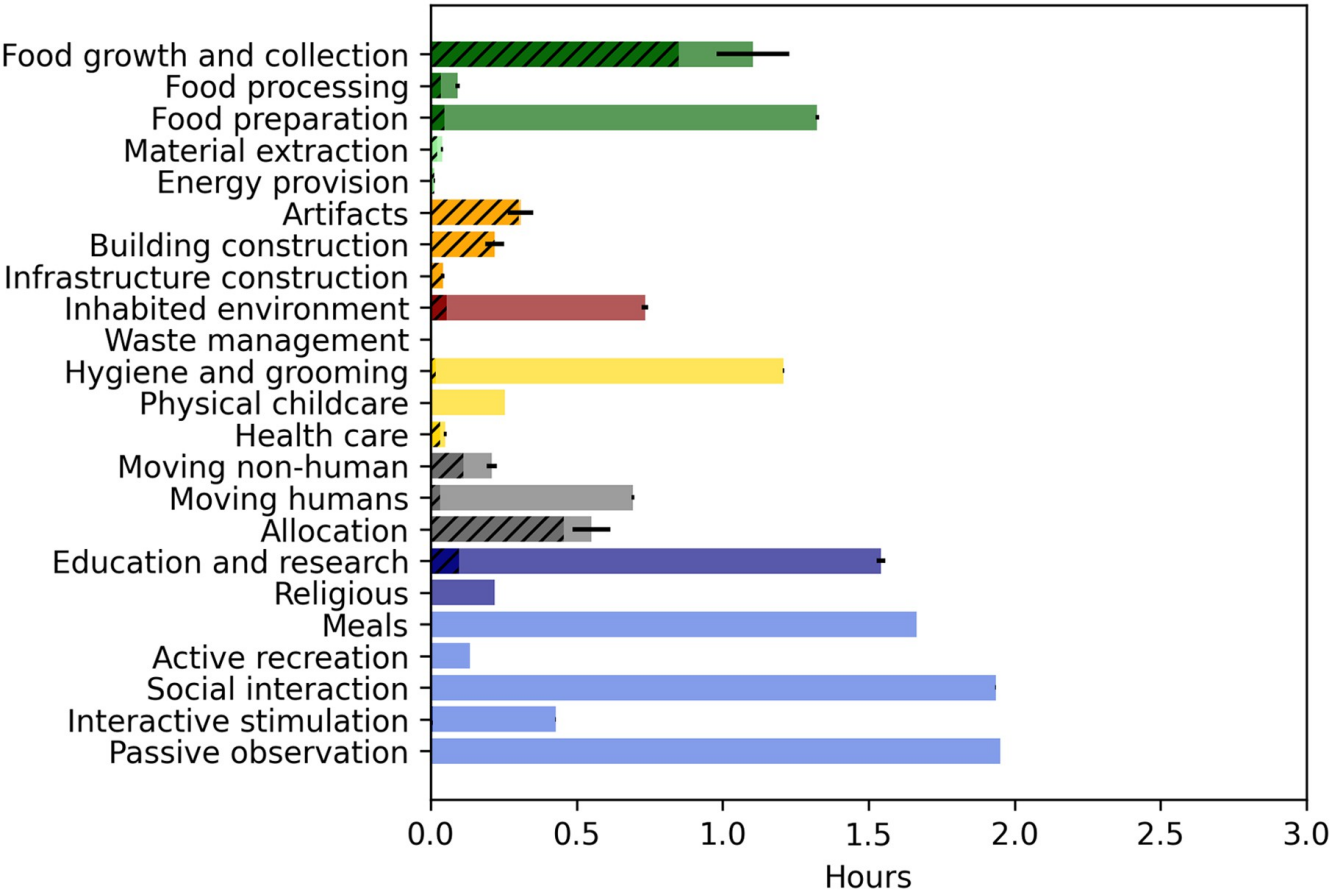
Time use in India using the MOOGAL. As in Fig 5.

Finally, we provide two example applications of the MOOGAL to time use data from specific domains. We choose Canada and India, given the large contrast in the resolution of their time use survey categories (*n* = 18 in Canada vs. *n* = 155 in India) as well as income levels. The Canadian data include the 2015 Time Use Survey [47] for non-paid work time and the 2019 economic data for paid work time [48, 49]. The Indian data include the 2019 Time Use Survey [50] for non-paid work time and the 2019 economic data for paid work time [51]. The results are shown in Figs 5 and 6.

In terms of the uncertainty introduced by the translation to the MOOGAL subcategories (indicated by error bars), the comparison shows very little additional uncertainty for the well-resolved Indian data, but considerably more for some categories of the highly-aggregated Canadian data. Primarily, the inclusion of all ‘housework’ as a single category in Canada leads to a large uncertainty in food preparation and inhabited environment maintenance.

Despite the coarse aggregation of the Canadian data, some differences in time use between the countries are clear. Although we do not dwell on these differences here, we note the much greater time in food growth and collection in India, greater time devoted to health care in Canada, greater time in hygiene and grooming in India, and much greater time in organizational activities (both transportation and allocation) in Canada. Given that the purpose of this comparison is simply to illustrate the potential of the method, we leave it to future work to explore differences such as these between populations, as revealed by consistent application of the MOOGAL.

## 7 Conclusion

Time use studies have long played a valuable role in social science research and policy. We suggest that the quantification of human status over time—the chronome—can gain much greater utility if more broadly unified through the use of parallel, generalized lexicons that embrace the multidimensionality of human states of being. This paper has identified two particular challenges to this aim: maintaining consistency in theoretical orientation, and the difficulty of dealing with the temporal and social aggregation of activities. To address these challenges we suggested that any generalized lexicon should be designed to follow a well-defined theoretical orientation and include specific protocols to minimize aggregational ambiguity, while complementary lexicons should be used in parallel to illuminate multiple dimensions of time use.

Toward this end, the paper has proposed a new general lexicon that reflects a compromise between theoretical aims and pragmatic usability, oriented along a dominant axis of variation among existing data: the MOOGAL. The use of a consistent theoretical orientation towards measurable physical outcomes is intended as a reasonably objective basis for categorizing activities, while the focus on motivating outcomes helps to avoid ambiguity, particularly for socially-aggregated activities. The MOOGAL follows a three-tiered priority scheme that reduces ambiguity related to multitasking and aggregation. The MOOGAL version here describes 8 high-level categories, encapsulating 24 finer-grained subcategories, which are in turn aligned with physical state variable classes that can be used to describe the human-Earth system. Our comparison between the MOOGAL and existing economic, sociological and anthropological lexicons revealed that the MOOGAL subcategories were able to capture most of the previously-described activities in a direct manner, indicating a useful alignment with these existing datasets, with very little ambiguity among high-level categories.

The version of the MOOGAL described here is intended as an imperfect first approach, subject to improvement. The lexicon could easily be modified in future to include finer-grained activities within its hierarchical structure. Because of its generalized nature, the MOOGAL could be easily adapted to use with new observations, such as those provided by digital devices (wearable technologies, digital media, etc.).

It is hoped that this generalized, physically-based approach will help illuminate the human chronome, further broadening the application of time use data to new fields of research, including the understanding of interactions between humans and the rest of the Earth system. There is currently great interest in re-evaluating how progress is measured [52], particularly given the aim of living well within planetary boundaries [53]. A better quantification of the global-scale human chronome can potentially make a central contribution to this end [40].

## Supporting information

S1 Fig Association of MOOGAL categories with CCSTA activities. Squares indicate the cross-mapping of hunter-gatherer activities to categories. Darkest shades indicate that an activity is entirely associated with a single MOOGAL category, paler shades indicate that the activity is distributed across multiple MOOGAL categories.
(PDF)

S2 Fig Association of MOOGAL categories with ISIC activities. Squares indicate the cross-mapping of hunter-gatherer activities to categories. Darkest shades indicate that an activity is entirely associated with a single MOOGAL category, paler shades indicate that the activity is distributed across multiple MOOGAL categories.
(PDF)

S3 Fig Association of MOOGAL categories with ICATUS activities, part one. Squares indicate the cross-mapping of hunter-gatherer activities to categories. Darkest shades indicate that an activity is entirely associated with a single MOOGAL category, paler shades indicate that the activity is distributed across multiple MOOGAL categories.
(PDF)

S4 Fig Association of MOOGAL categories with ICATUS activities, part two. Squares indicate the cross-mapping of hunter-gatherer activities to categories. Darkest shades indicate that an activity is entirely associated with a single MOOGAL category, paler shades indicate that the activity is distributed across multiple MOOGAL categories.
(PDF)

S1 Data (ZIP)
