# Supplementary figures and images for: Interdisciplinary applications of human time use with generalized lexicons
Source: PLoS One. 2022 Jul 14;17(7):e0270583. doi: 10.1371/journal.pone.0270583 (PMC9282456; doi:10.1371/journal.pone.0270583)

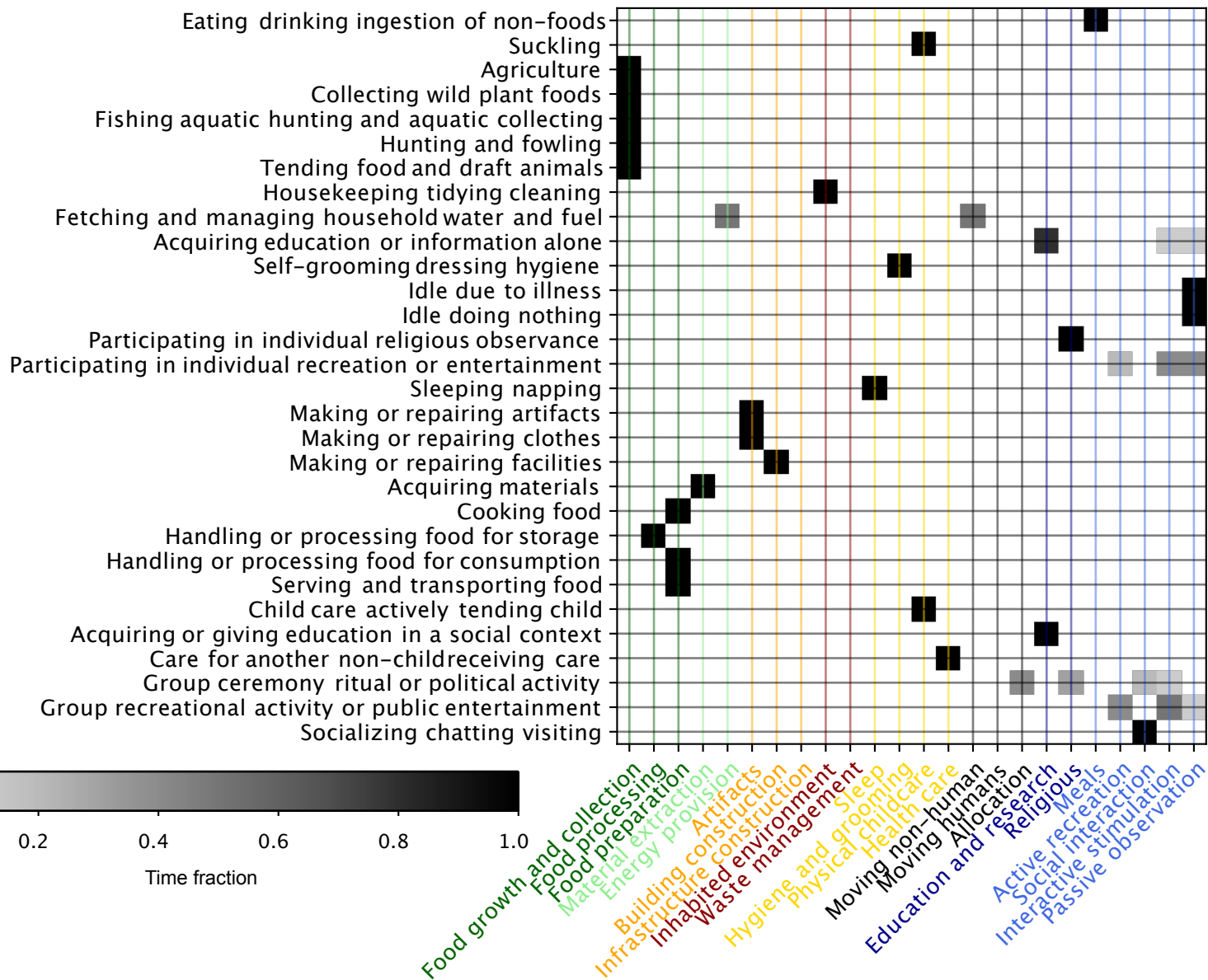

Supplement: S1 Fig — Squares indicate the cross-mapping of hunter-gatherer activities to categories. Darkest shades indicate that an activity is entirely associated with a single MOOGAL category, paler shades indicate that the activity is distributed across multiple MOOGAL categories. (PDF) [file pone.0270583.s001.pdf]

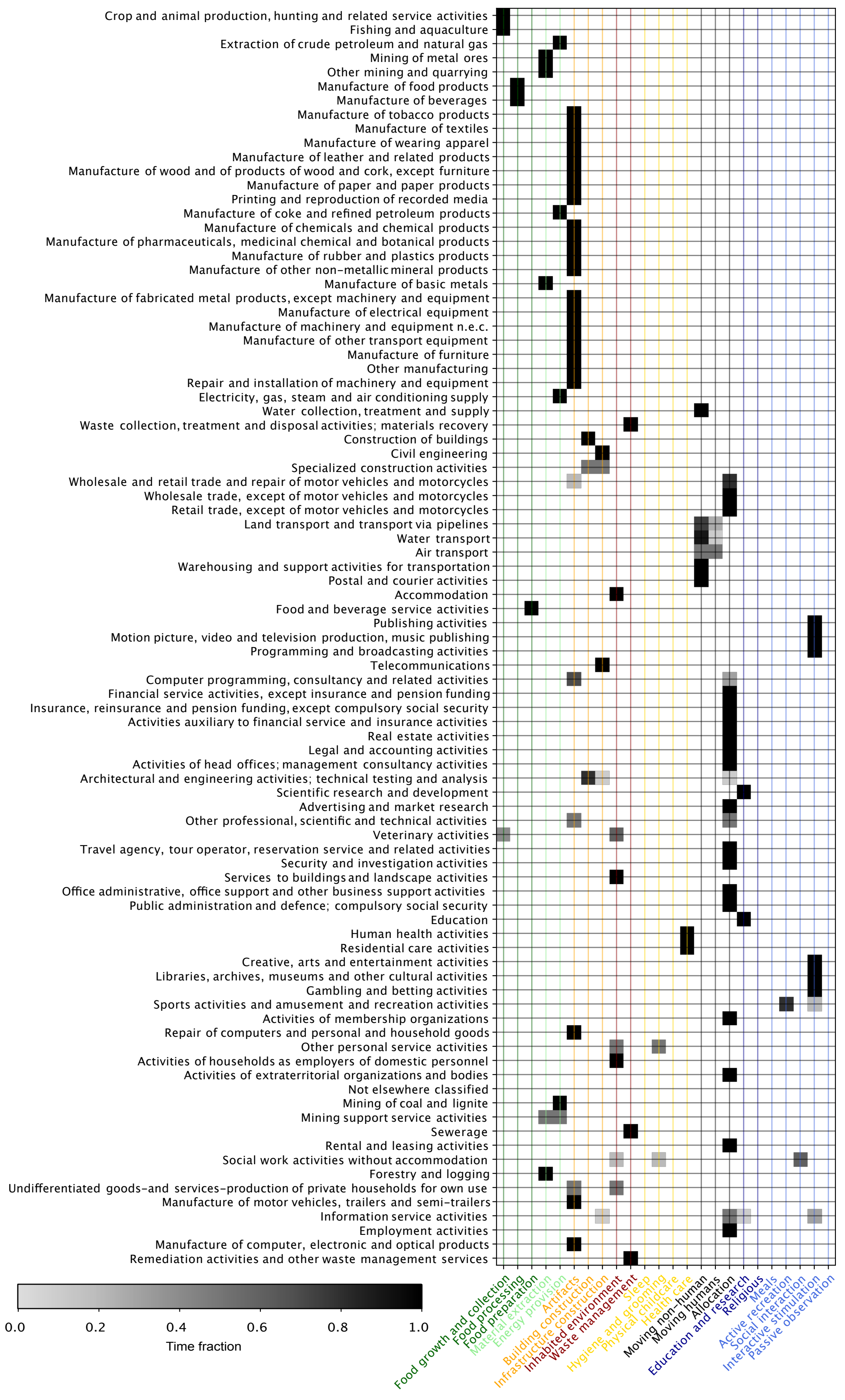

Supplement: S2 Fig — Squares indicate the cross-mapping of hunter-gatherer activities to categories. Darkest shades indicate that an activity is entirely associated with a single MOOGAL category, paler shades indicate that the activity is distributed across multiple MOOGAL categories. (PDF) [file pone.0270583.s002.pdf]

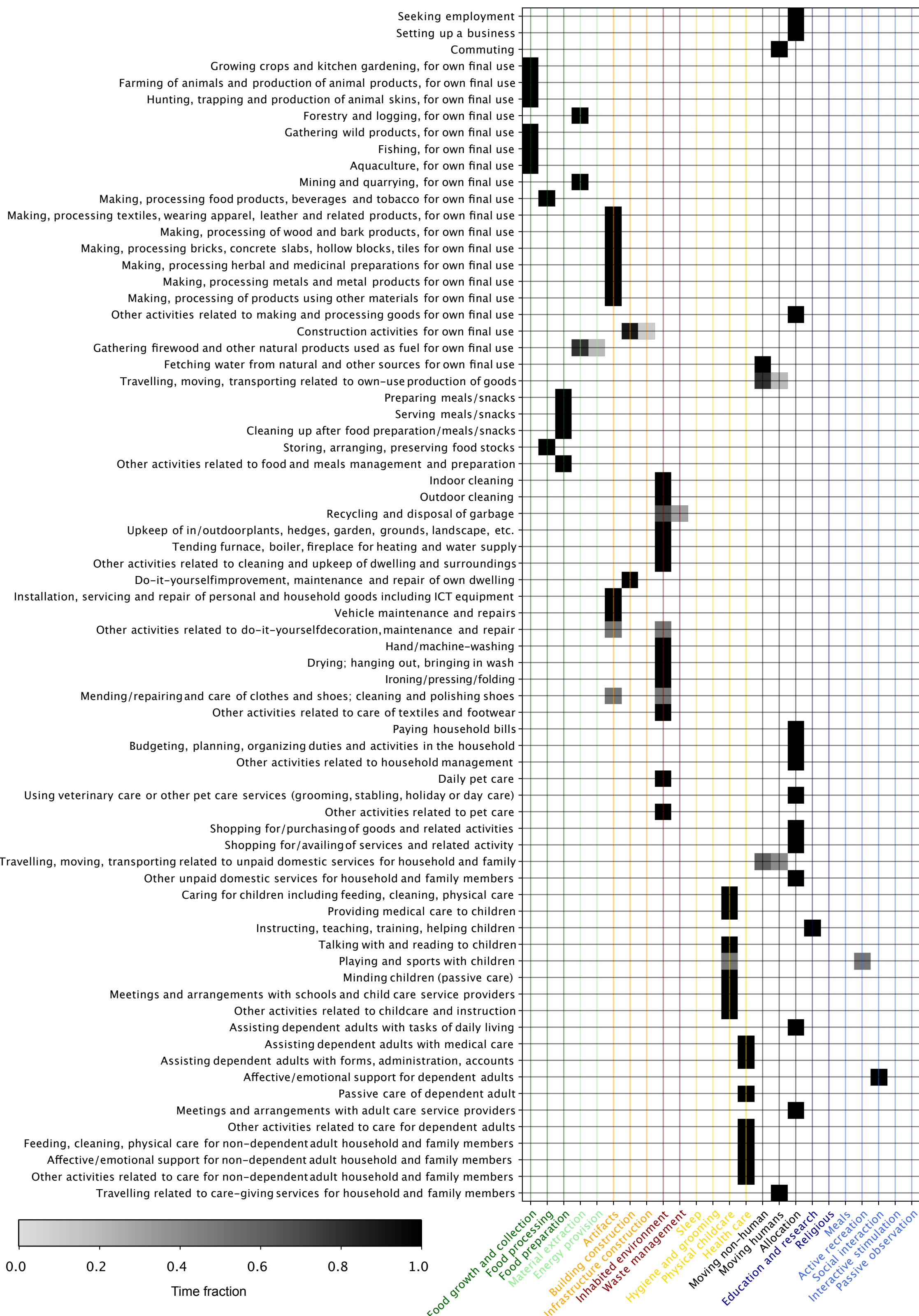

Supplement: S3 Fig — Squares indicate the cross-mapping of hunter-gatherer activities to categories. Darkest shades indicate that an activity is entirely associated with a single MOOGAL category, paler shades indicate that the activity is distributed across multiple MOOGAL categories. (PDF) [file pone.0270583.s003.pdf]

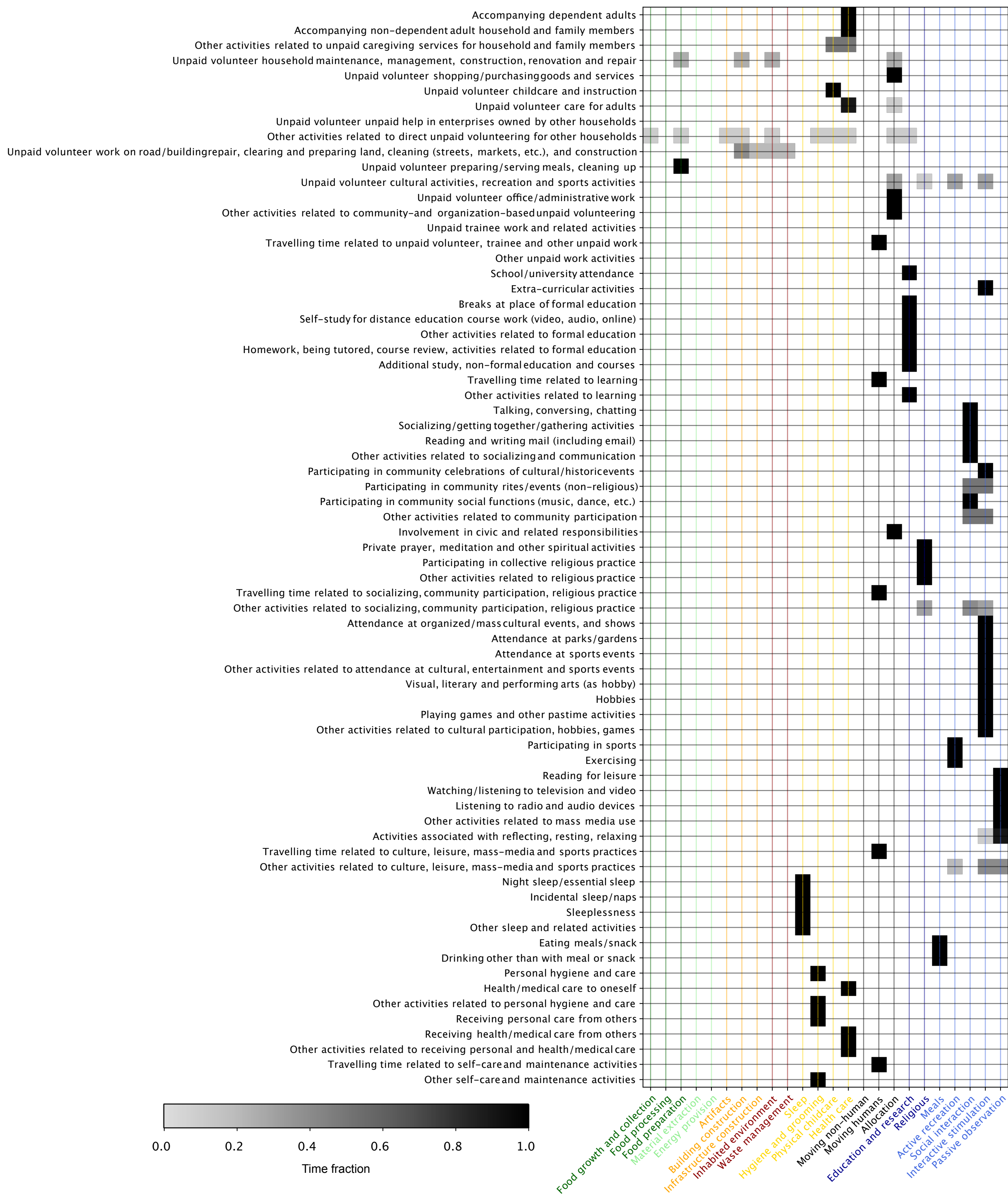

Supplement: S4 Fig — Squares indicate the cross-mapping of hunter-gatherer activities to categories. Darkest shades indicate that an activity is entirely associated with a single MOOGAL category, paler shades indicate that the activity is distributed across multiple MOOGAL categories. (PDF) [file pone.0270583.s004.pdf]
